# Determinants of poor glycemic control among type 2 diabetes in Ethiopia: a systematic review and meta-analysis

**DOI:** 10.3389/fpubh.2024.1256024

**Published:** 2024-02-05

**Authors:** Kirubel Dagnaw Tegegne, Natnael Atnafu Gebeyehu, Lehulu Tilahun Yirdaw, Yibeltal Asmamaw Yitayew, Mesfin Wudu Kassaw

**Affiliations:** ^1^Department of Nursing, College of Medicine and Health Science, Wollo University, Dessie, Ethiopia; ^2^Department of Midwifery, College of Medicine and Health Science, Wolaita Sodo University, Sodo, Ethiopia; ^3^Department of Emergency Nursing, College of Medicine and Health Science, Wollo University, Dessie, Ethiopia; ^4^Department of Pediatrics and Child Health Nursing, College of Medicine and Health Science, Wollo University, Dessie, Ethiopia; ^5^School of Nursing, College of Health Science, Woldia University, Woldia, Ethiopia

**Keywords:** poor glycemic control, determinants, meta-analysis, type 2 diabetes, Ethiopia

## Abstract

**Introduction:**

Prior primary studies have examined the prevalence and factors associated with glycaemic control among patients with type 2 diabetes mellitus, but studies with evidence-based synthesis of the primary data remained unknown. Hence, we aimed to determine the prevalence of poor glycemic control and identify determinants of poor glycemic control in patients with type 2 diabetes in Ethiopia.

**Methods:**

We performed searches in the online databases of PubMed, Google Scholar, Scopus, Science Direct, and the Cochrane Library. Microsoft Excel was used to extract data, and STATA statistical software (v. 16) was used for analysis. Publication bias was explored by forest plots, Begg’s rank test, and Egger’s regression test. To check for heterogeneity, I^2^ was computed. Subgroup analysis was conducted based on region and publication year. In addition, the pooled odds ratio for associated factors was calculated.

**Results:**

Out of 1,045 studies assessed, 23 studies were included fulfilling our inclusion criteria. In all, 6,643 individuals were enrolled in the study. It was estimated that 61.11% of type 2 diabetes patients had poor glycemic control (95% CI, 57.14–65.19). The subgroup analysis by study region and publication year revealed that the highest prevalence was observed in the Addis Ababa region (68.57%) and studies published before 2019 (61.76%), respectively. Poor glycemic control was associated with older age > 50 years (AOR = 2.12; 95% CI: 1.27–2.97), not attending formal education (AOR = 3.60; 95% CI: 2.75, 4.46), having diabetes for longer duration (10 years; AOR = 2.57; 95% CI: 1.65–3.49), having comorbidity (AOR = 2.43; 95% CI: 2.05–2.80), and low adherence to diabetes management (AOR = 3.67; 95% CI: 2.41–4.92).

**Conclusion:**

Our findings indicate a high prevalence of poor glycemic control among people with type 2 diabetes in Ethiopia. Being older, not attending formal education, having a longer duration of diabetes, having comorbidity, and having low adherence to diabetes management were all associated. Therefore, we recommend health organizations implement measures to monitor and control patients’ blood glucose levels. Patient education and training of healthcare professionals could serve as a short-term strategy to achieve adequate glycemic control.

**Systematic review registration:**

PROSPERO, identifier CRD42022349792, https://www.crd.york.ac.uk/prospero/display_record.php?ID=CRD42022349792.

## Background

Diabetes mellitus (DM) is a rapidly rising global health challenge with a huge burden of diseases (1). It is a common metabolic disorder caused by either insufficient insulin secretion from the pancreas, reduced insulin action, or both (2). The latest data show that in 2019, 463 million individuals are affected by diabetes worldwide. This number is projected to increase to 578 million patients by 2030 and further rise to 700 million by 2045 (3). The estimated number of adults living with diabetes in sub-Saharan Africa (SSA) in 2017 was 15.5 million. Reports found that approximately 69% of SSA adults living with diabetes remained undiagnosed (3, 4). Ethiopia has recorded 2.6 million diabetic patients, the highest number of diabetes cases in Africa (5). Therefore, effective management is the priority for these patients in Ethiopia.

Type 2 diabetes mellitus (T2DM) is characterized by the inability of the pancreas to secrete insulin and peripheral insulin resistance (2). T2D represents 90–95% of all diabetes cases. Despite increased global attention and healthcare efforts, its prevalence continues to increase (6). Nowadays, new methods of evaluating glycemic control are underway. Inadequate diabetes management and its complications can result in recurrent hospital admissions and premature death. Glycemic control plays a key role in the prognosis of diabetes and possible complications (7). A reasonable glycated hemoglobin (A1C) goal for glycemic control is 7%. However, less stringent (A1C of 8%) or more stringent (e.g., A1C of 6.5%) goals might be considered for some patients (2).

Despite the observed benefits of adequate glycemic control, evidence suggests that the majority of patients did not meet targets for reasonable HbA1C according to the recommendations of consensus treatment panels. According to combined regional estimates of North Africa and the Middle East, only approximately 37% of the patients with T2D had an HbA1C < 7.0% in 2021 (8). Furthermore, studies have also suggested that glycemic control in Ethiopia is suboptimal, which indicates most patients (65.5%) had poor glycemic control (9).

Previous meta-analyses of glycemic control in Ethiopia have provided a comprehensive report on all diabetes patients (9). However, a meta-analysis of factors of poor glycemic control in T2DM is still lacking. In Ethiopia, several individual studies have investigated the level of glycemic control and its associated factors among T2D patients. However, most of these studies are single-centered and have relatively small sample sizes. Thus, systematic review and meta-analysis are required, as they provide a comprehensive overview of glycemic control in T2D as a country-level burden. Estimating the national level of glycemic control and identifying associated factors is invaluable to inform strategy design and policy-making to mitigate the burden of T2D. Therefore, this systematic review and meta-analysis aimed to evaluate the level and factors of poor glycemic control among T2D patients in Ethiopia.

## Methods

We prepared and presented this study according to Preferred Reporting Items for Systematic Reviews and Meta-Analyses (PRISMA; Supplementary Table S1) (10) and registered on PROSPERO under a registration number of CRD42022349792.

### Search strategy

We identified potentially eligible studies by systematically searching the databases of PubMed, Science Direct, Scopus, and Google Scholar. The keywords used during the search were: ((((((((((((((Glycemic control) OR (poor glycemic control)) OR (blood glucose)) OR (Hemoglobin A1C)) OR (HBA1C)) AND (Diabetes mellitus)) OR (Type 2)) OR (Type II diabetes)) OR (Type 2 diabetes)) AND (associated factors)) OR (determinants)) OR (predictors))) AND (Adult)) AND (Ethiopia). Search terms were based on PICO principles to retrieve relevant articles through the databases mentioned above. We have searched for articles from 1 May 2022 to 30 May 2022.

### Outcome measurement

Patients with an FPG level of ≤130 mg/dL or an A1C level of ≤7% were considered to have good glycemic control. On the other hand, patients with an FPG level of *>*130 mg/dL or an A1C level of *>*7% were considered to have poor glycemic control (2).

### Eligibility criteria

This meta-analysis includes studies that reported the level of glycemic control in adult T2D as study participants, published in English as full-length articles, both published and unpublished studies with full text available for search, and studies that took place in Ethiopia. Studies published between 1 January 2000 and 30 May 2022 were included. We excluded duplicated studies, conference papers or abstracts, articles without full texts, and studies with data that could not be obtained from the corresponding authors.

### Quality appraisal

A set of standardized critical appraisal instruments from the Joanna Briggs Institute for observational studies was used to evaluate the quality of the included studies (11). The instrument assesses whether the original studies provided adequate information regarding population characteristics, exposures, confounders, outcomes, follow-up details (where applicable), and statistical analysis. The answers to each instrument item were labeled as yes, no, unclear, or not applicable. Any inconsistencies were resolved through discussion led by the third author (GAA). The critical appraisal tool is shown in Supplementary Table S2.

### Data extraction

Using a standard data extraction form, two investigators (KDT and NAG) independently extracted the following information from the included studies. This information includes the name of the first author, year of publication, study region, study setting, study design, the prevalence of poor glycemic control, sample size, and quality of each study. Any disagreements that arose during data extraction were resolved through a discussion led by the third author (LTY). Finally, the argument was solved and an agreement reached. The data automation tool was not used due to this study’s absence of the paper form (manual data).

### Data analysis

After extracting all relevant findings in a Microsoft Excel spreadsheet, the data were exported into STATA software version 16 for analysis. Random effect models (DerSimonian and Laird methods) were used to pool individual studies. The possibility of publication bias was explored by the visual inspection of the funnel plots and more objectively by Begg’s and the Egger test (12) and a value of *p* less than 0.05 indicates it is statistically significant (13). The presence of between-study heterogeneity was checked by using the Cochrane Q statistic. Between studies, heterogeneity was investigated using I^2^, in which a value of 25, 50, and 75% represented low, medium, and high heterogeneity, respectively (14). To identify the subgroup differences and potential sources of the observed heterogeneity, we carried out subgroup analysis based on the study region and publication year (before 2019 and 2019 onwards). To evaluate the robustness of the overall estimates, we performed sensitivity analyses, leaving out each study consecutively and observing any notable changes in the pooled result. All statistical analyses were conducted in STATA 14.0 (Stata Corp., College Station, TX, United States).

## Results

### Study selection

**Figure 1:**
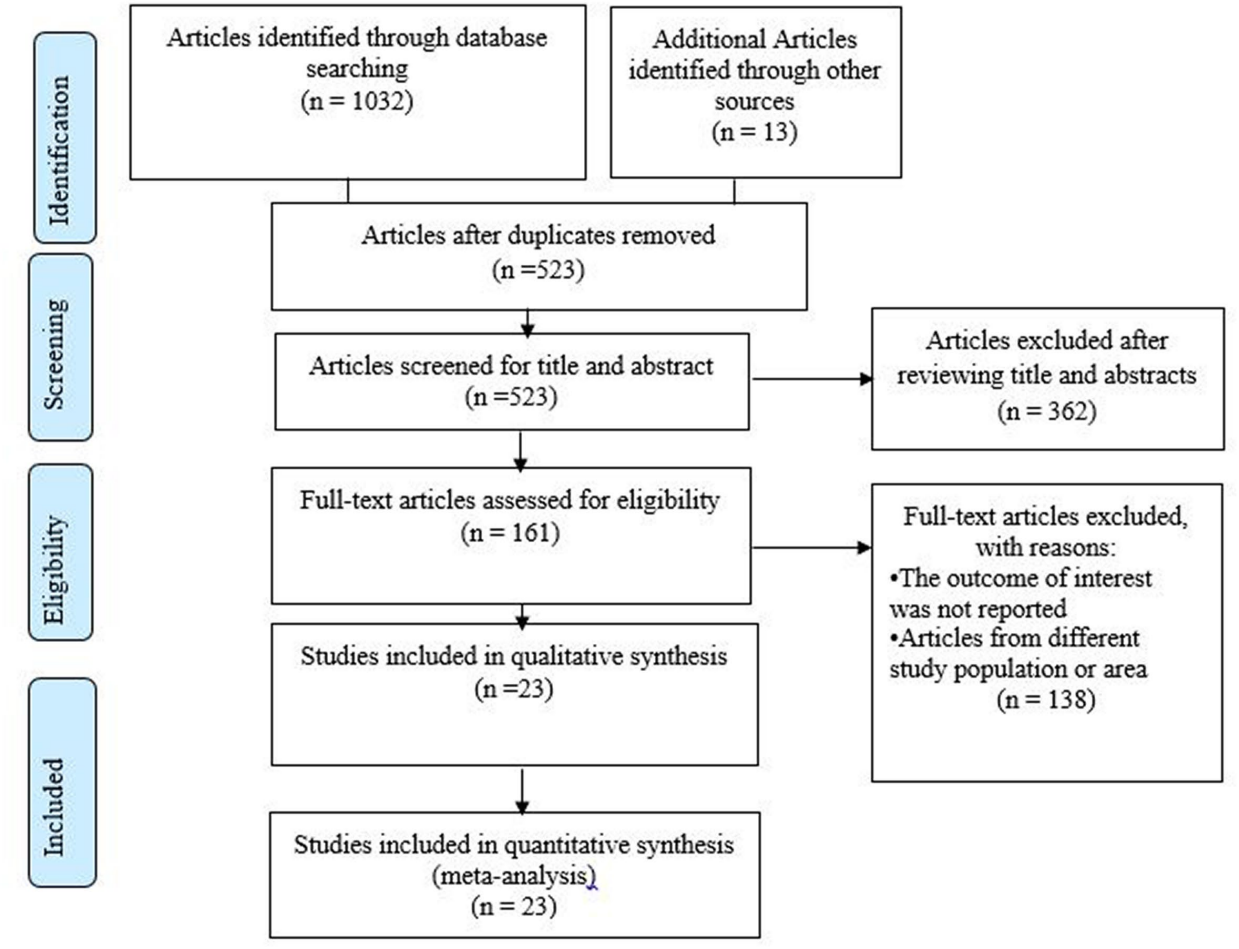
Flow chart illustrating the process of search and selection of studies included in the present systematic review and meta-analysis.

We identified 1,045 records through the electronic databases of PubMed, Scopus, Google Scholar, ScienceDirect, and the Cochrane library. Removing duplicates resulted in 523 articles. Then, full title and abstract screening excluded 362 studies, and 523 studies remained. Therefore, 161 full-text studies were assessed for eligibility, which further excluded 138 studies due to the lack of reported outcomes of interest and studies other than the study area. Finally, 23 articles were included in the systematic review and meta-analysis (Figure 1).

### Characteristics of included studies

A total of 23 studies, including 6,643 participants, were identified. The included studies were published between 2011 and 2022. All studies were institutional observational studies, of which 21 were cross-sectional study designs, 1 was a retrospective cohort, and 1 was a case–control. Ten studies were conducted in the Oromia region (7, 15–23), five in Addis Ababa (24–28), four in Amhara (29–32), two in Tigray (33, 34), and two in Dire Dawa (35, 36). The sample sizes ranged from 102 to 412. The prevalence of poor glycemic control ranges from 41.36 to 78.64. Selected articles were deemed to have satisfactory methodological quality as per the critical appraisal process (Table 1).

**Table 1:** Summary characteristics of studies included in the meta-analysis.

Author	Year	Region	Study design	Sample size	Mean age	Mean duration of T2DM in year	Mean blood glucose	Prevalence	Diagnostic criteria	Quality
Fasil. A et al.	2019	Amhara	Cross-sectional	367	48.6	Not stated	Not stated	59.8	FPG > 154 mg/dL	Low-risk
Gebremariam AD. et al.	2020	Amhara	Cross-sectional	398	52.6	5.32	175.52 mg/dL	71.35	FPG > 130 mg/dL	Low-risk
Mohammed AS. et al.	2020	Dire Dawa	Cross-sectional	307	Not stated	6,37	154.2 mg/dL	54.72	FPG > 130 mg/dL	Low-risk
Ayele AA. et al.	2019	Amhara	Cross-sectional	275	52.7	6.08	161.4 mg/dL	57.1	FPG > 130 mg/dL	Low-risk
Bayisa B. et al.	2017	Oromia	Cross-sectional	174	50.18	2.19	130.38 mg/dL	63.8	FPG > 130 mg/dL	Low-risk
Fseha B. et al.	2017	Tigray	Cross-sectional	200	42.2	Not stated	Not stated	63.5	FPG > 130 mg/dL	Low-risk
Abdissa D. et al.	2021	Oromia	Cross-sectional	279	46.45	Not stated	Not stated	65.6	A1C ≥ 7	Low-risk
Yigazu DM. et al.	2017	Oromia	Cross-sectional	174	49.98	Not stated	130.3 mg/dL	59.2	FPG > 130 mg/dL	Low-risk
Demoz GT. et al.	2019	Addis Ababa	Cross-sectional	357	56	11.64	174.1 mg/dL	68.34	FPG > 130 mg/dL	Low-risk
Fekadu G. et al.	2019	Oromia	Cross-sectional	228	43	Not stated	Not stated	64.91	FPG > 130 mg/dL	Low-risk
Bereda G. et al.	2021	Oromia	Cross-sectional	122	Not stated	Not stated	Not stated	60.65	FPG > 130 mg/dL	Low-risk
Woldu MA. et al.	2014	Oromia	Cross-sectional	102	51.75	4.0	168.7 mg/dL	50.0	FPG > 126 mg/dL	Moderate-risk
Wabe NT. et al.	2011	Oromia	Cross-sectional	384	48.3	Not stated	Not stated	58.07	FPG > 100 mg/dL	Low-risk
Shita NG. et al.	2022	Amhara	Retrospective cohort	191	57.9	3.6	137.35 mg/dL	41.36	FPG > 130 mg/dL	Low-risk
Abera RG. et al.	2022	Addis Ababa	Cross-sectional	325	Not stated	9	A1C-8.4%	56.92	A1C ≥ 7	Low-risk
Nigussie S. et al.	2021	Dire Dawa	Cross-sectional	394	40.76	8.93	154.57 mg/dL	45.17	FPG > 154 mg/dL	Low-risk
Eticha T. et al.	2016	Tigray	cross-sectional	384	52.8	6.9	Not stated	48.7	A1C ≥ 7	Low-risk
Alemu T. et al.	2021	Addis Ababa	Cross-sectional	245	49.55	Not stated	Not stated	77.95	FPG > 125 mg/dL	Low-risk
Shimels T. et al.	2018	Addis Ababa	Cross-sectional	361	54.8	6.7	154 mg/dL	60.66	FPG > 130 mg/dL	Low-risk
Kassahun T. et al.	2016	Oromia	cross-sectional	309	Not stated	7.2	Not stated	70.87	FPG > 130 mg/dL	Low-risk
Yosef T. et al.	2021	Oromia	cross-sectional	245	48.6	Not stated	Not stated	64.08	FPG > 130 mg/dL	Low-risk
Tekalegn Y. et al.	2018	Oromia	cross-sectional	412	Not stated	10	165.63 mg/dL	78.64	FPG > 130 mg/dL	Low risk
Mamo Y. et al.	2019	Oromia	Case–control	410	52.63	Not stated	Not stated	–	FPG > 130 mg/dL	Low risk

A1C-glycated hemoglobin, FPG-fasting blood glucose.

### Glycemic control

Glycemic control was evaluated commonly based on fasting blood glucose (FBG), as reported in 20 studies (7, 16–23, 25–32, 34–36), whereas only 3 studies assessed glycemic control using HbA1C (15, 24, 33). Fifteen studies considered poor glycemic control above 130 mg/dL (7, 16–19, 22, 23, 26–29, 31, 32, 34, 35), three studies considered it at 126 mg/dL (20, 21, 25), and two studies defined poor glycemic control as higher than 154 mg/dL (30, 36). As of the level of A1C, three studies considered poor glycemic control at A1C ≥ 7% (15, 24, 33). A total of 11 studies reported participants’ mean duration of T2D starting from diagnosis, and based on these studies, the mean duration of T2D was 7.42 years. Similarly, the mean FBG level was reported in 11 studies, and the overall mean FBG was 155.08 mg/dL.

## Meta-analysis

### Prevalence of poor glycemic control among T2D in Ethiopia

**Figure 2:**
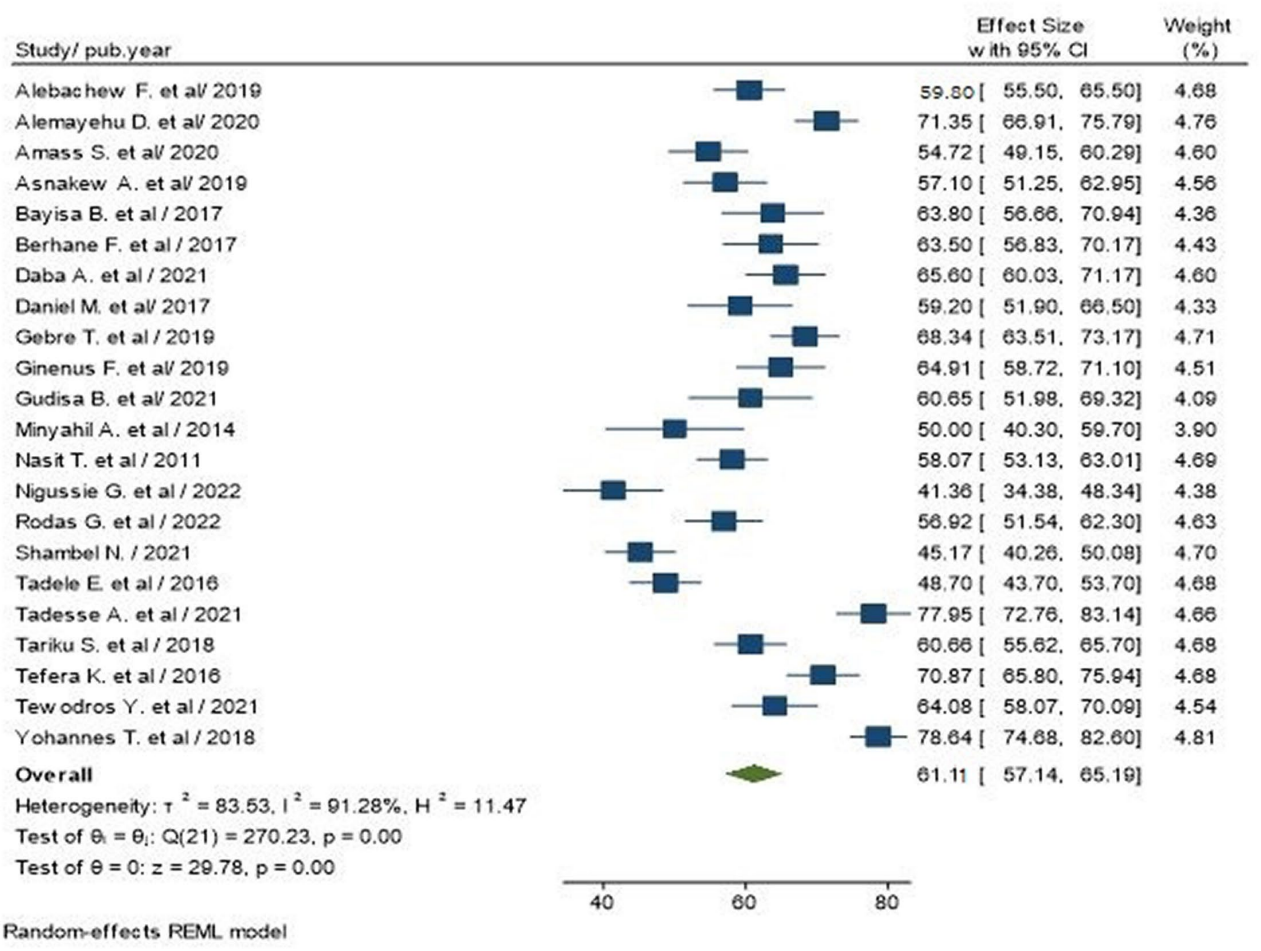
Pooled prevalence of poor glycemic control among type 2 diabetic people in Ethiopia.

Out of the 23 studies included in our review, 1 study did not report the prevalence estimates. Thus, 22 studies, including 6,233 participants, reported the prevalence of poor glycemic control, ranging from 41.36 to 78.64%. The pooled prevalence of poor glycemic control among T2D in Ethiopia was 61.11% (95% CI: 57.14–65.19; I^2^ = 91.28%, *p* < 0.00; Figure 2).

### Subgroup analysis

A subgroup analysis was conducted to observe variations across potential covariates and explore the sources of heterogeneity. As a result, subgroup analysis was conducted in terms of study region and publication year. In terms of region, the highest prevalence of poor glycemic control was observed in Addis Ababa (68.57%), and subgroup analysis by publication year revealed a higher pooled prevalence of 61.76% across studies published before 2019 (Table 2).

**Table 2:** Subgroup analysis of studies included in the meta-analysis on poor glycemic control and associated factors among T2D in Ethiopia.

Subgroup	Random effects (95% CI)	Test of heterogeneity (I^2^) (%)
By region		
Amhara	57.77% (45.71–69.84%)	94.91
Oromia	62.64% (58.98–66.11%)	63.66
Addis Ababa	68.57% (59.95–77.19%)	93.72
Dire Dawa	49.86% (40.49–69.21%)	84.26
Tigray	55.93% (41.43–70.43%)	91.74
Publication year		
Before 2019	61.76% (55.51–68.01%)	90.88
2019 and after	60.75% (55.3–66.21%)	92.03

### Meta regression

We have performed meta-regression to further explore sources of heterogeneity among studies. A meta-regression using publication year and sample size as covariates indicated non-significant results. The results of meta-regression are presented in Table 3.

**Table 3:** Meta-regression analysis of factors affecting between-study heterogeneity.

Covariate	Coefficients	Std. Err.	*p* value
Publication year	0.0749	0.8013	0.926
Sample size	0.0160	0.0234	0.495

### Publication bias

**Figure 3:**
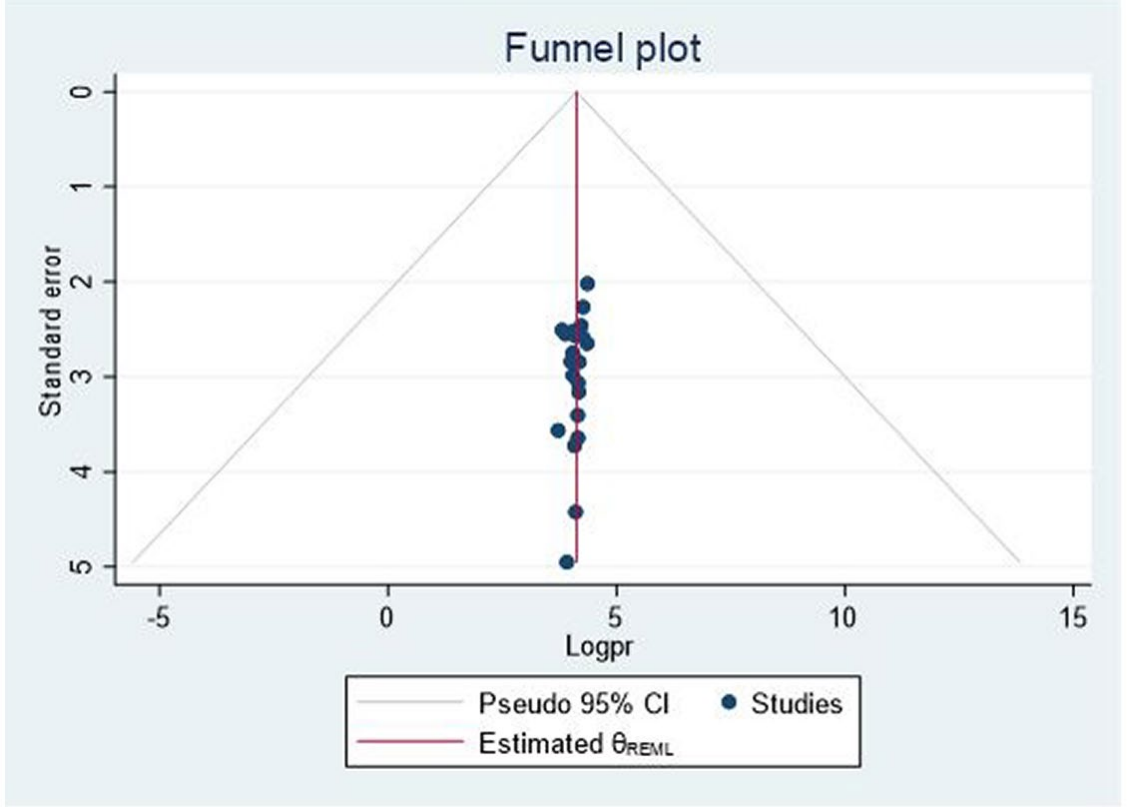
Funnel plot, which shows the symmetrical distribution of studies.

Visual inspection of the funnel plot indicated no evidence of publication bias. Further investigation of Egger’s (*p* = 0.0783) and Begg’s regression tests (*p* = 1.7637) did not confirm the presence of publication bias (Figure 3).

### Sensitivity analysis for the studies included

**Figure 4:**
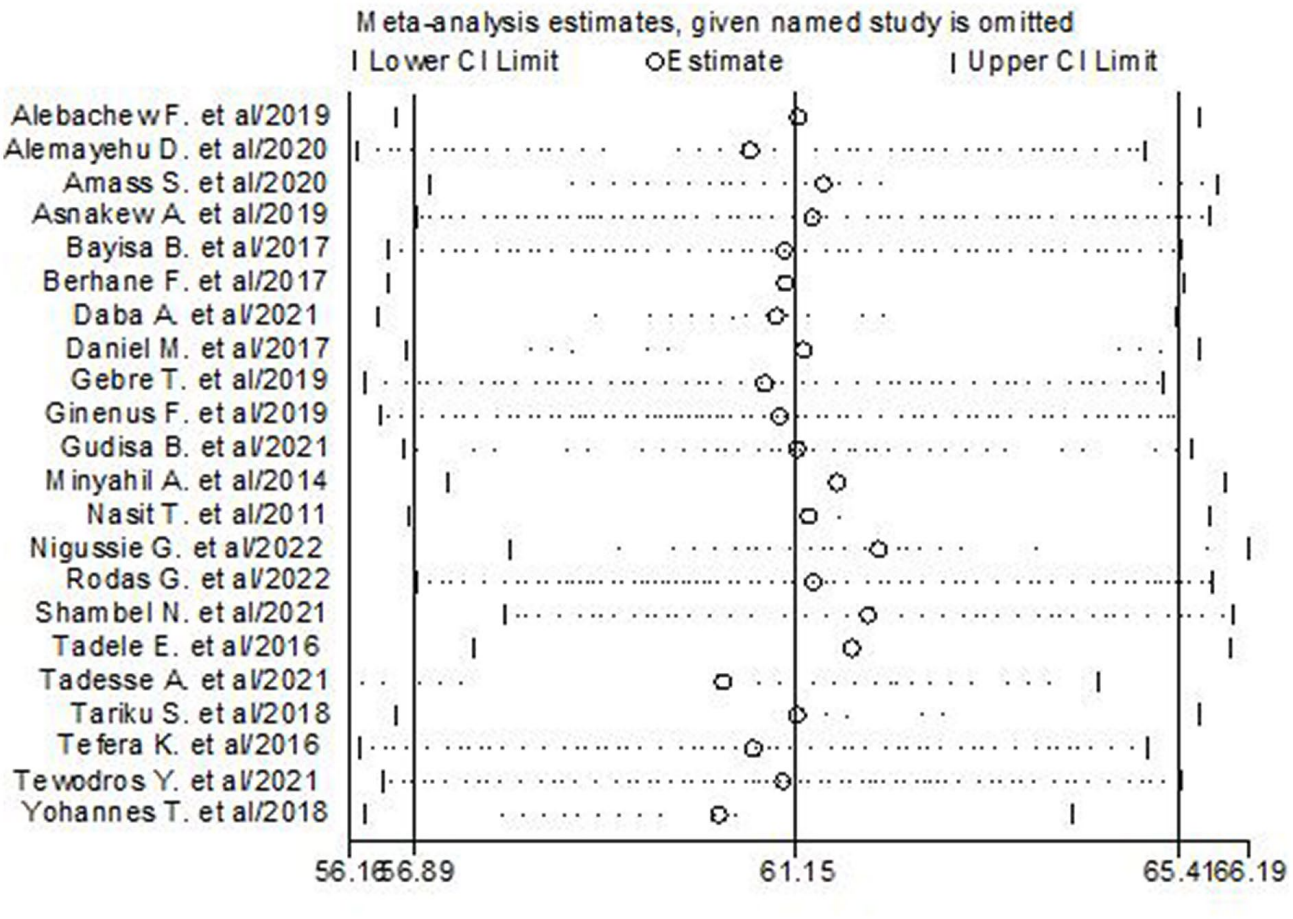
Results of the sensitivity of the 22 studies in the meta-analysis of glycemic control in T2DM.

To examine the effect of a single study on the overall prevalence estimates, sensitivity analysis was performed using a random effect model. The result revealed that the overall estimate was not affected when studies were alternatively omitted from the model. The pooled estimated prevalence of poor glycemic control is estimated to range from 60.29 (56.35–64.22) to 62.07 (57.94–66.19) after the omission of a single study (Figure 4).

### Factors associated with poor glycemic control among T2D

In this systematic review and meta-analysis, factors that are associated with poor glycemic control in T2D patients have been identified. Subsequently, older people >50 years, not attending formal education, longer duration of diabetes (10 years), having comorbidity, and low adherence to diabetes management are the factors linked with poor glycemic control among T2DM patients. However, the combined use of oral anti-diabetics and oral insulin was not a significant factor in poor glycemic control among T2D patients.

### Association of older age with poor glycemic control

**Figure 5:**
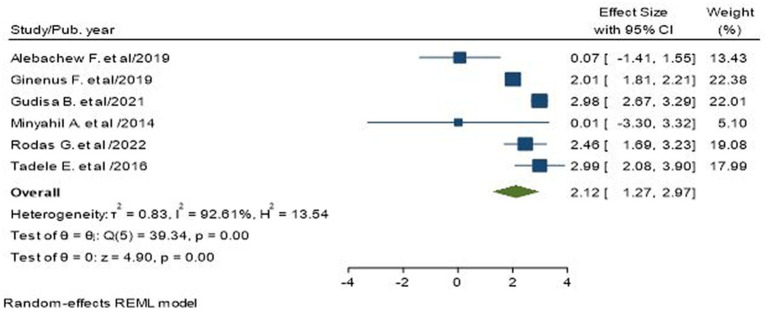
Association between older age and poor glycemic control in type 2 diabetic patients.

Six studies, including 1,528 participants, provided data for this analysis (17, 18, 21, 24, 30, 33). Accordingly, older age (over 50 years) was found to be a factor associated with poor glycemic control. The pooled result indicated that older people >50 years were two times more likely to have poor glycemic control than younger people (AOR = 2.12; 95% CI: 1.27, 2.97; I^2^ = 92.61%, *p* < 0.001; Figure 5).

### Association of educational status with poor glycemic control

**Figure 6:**
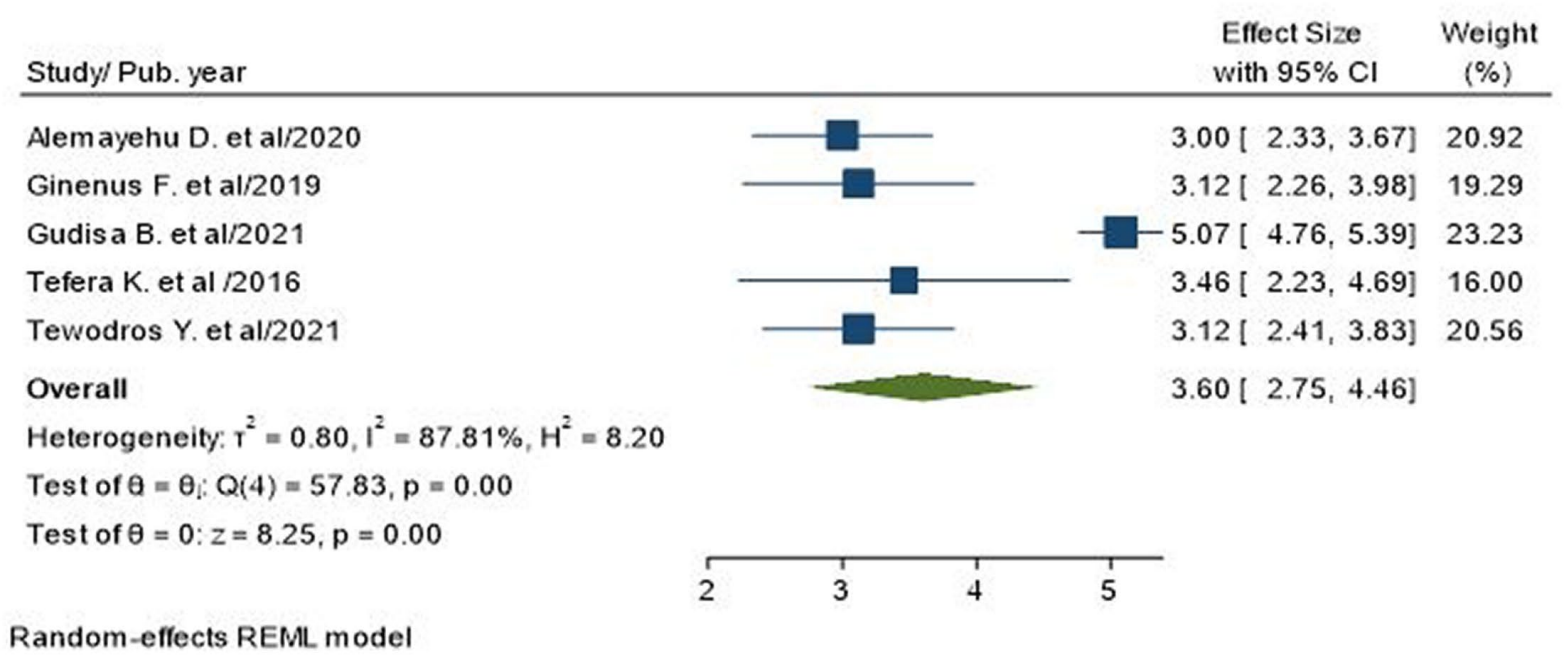
Association between educational status and poor glycemic control in type 2 diabetic patients.

Educational status, more specifically, not attending formal education, was reported as a significant factor influencing glycemic control among T2D patients in four primary studies (17–19, 23, 31). A total of 1,302 subjects were included to analyze the association between not attending formal education and poor glycemic control. The odds of poor glycemic control among uneducated type 2 diabetics were 3.6 times higher as compared to their educated counterparts (AOR = 3.60; 95% CI: 2.75, 4.46; I^2^ = 87.81%, *p* < 0.001; Figure 6).

### Association of longer duration of diabetes with poor glycemic control

**Figure 7:**
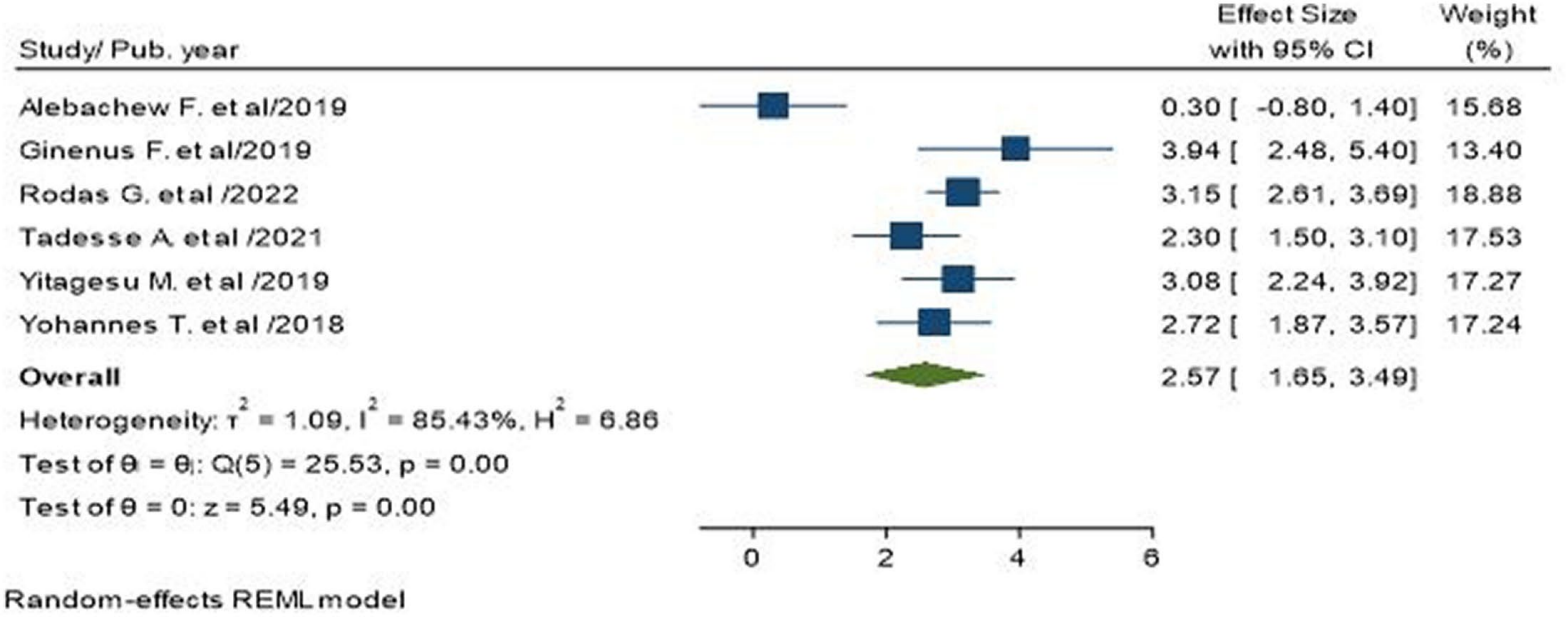
Association between longer duration of diabetes and poor glycemic control in type 2 diabetic patients.

Six primary studies, involving a total of 1987 subjects, reported longer duration with diabetes (10 years) as a factor for poor glycemic control in T2D patients (7, 18, 24, 25, 28, 30). The pooled odds ratio showed that longer duration of T2D was 2.57 times more likely to have poor blood glucose control than their counterparts (AOR = 2.57; 95% CI: 1.65–3.49; I^2^ = 85.43%, *p* < 0.001; Figure 7).

### Association of comorbidity with poor glycemic control

**Figure 8:**
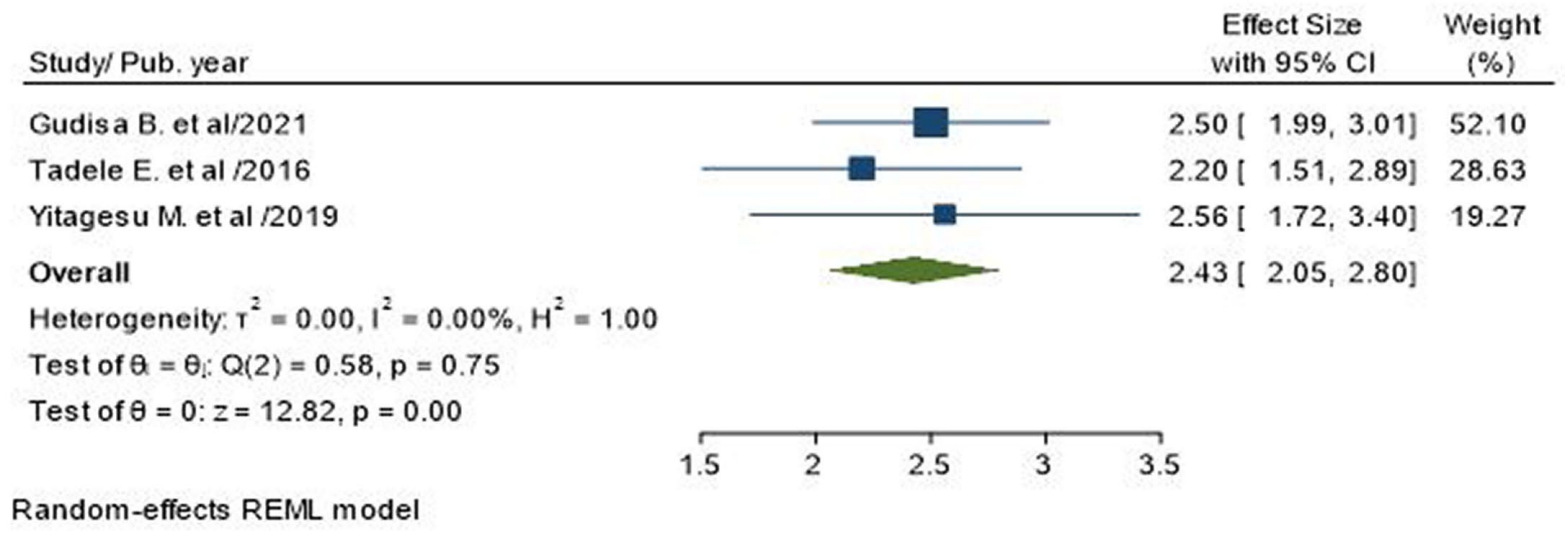
Association of combined use of oral anti-diabetics and insulin with poor glycemic control in type 2 diabetic patients.

Three studies, involving a total of 916 subjects, reported longer duration with diabetes (10 years) as a factor for poor glycemic control in T2DM patients (7, 17, 33). The pooled odds ratio showed that T2D with comorbidity was 2.43 times more likely to have poor blood glucose control than T2D without comorbidity (AOR = 2.43; 95% CI: 2.05–2.80; I^2^ = 0.00%, *p* < 0.001; Figure 8).

### Association of low self-care adherence with poor glycemic control

**Figure 9:**
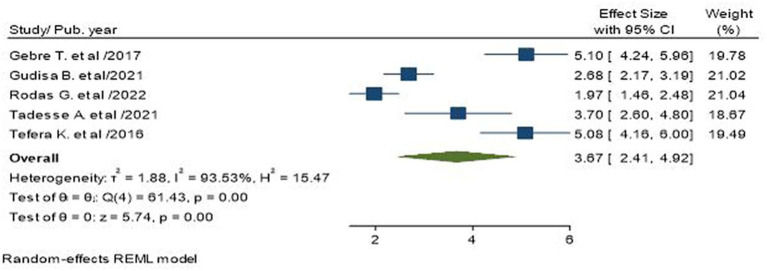
Association between low self-care adherence and poor glycemic control in type 2 diabetic patients.

Five studies, including 1,358 study subjects, reported low self-care adherence as a factor in poor glycemic control in T2D patients (17, 19, 24–26). The pooled odds ratio showed that T2D with low self-care adherence had 3.67 times more odds to control blood glucose than T2D with good self-care adherence (AOR = 3.67; 95% CI: 2.41–4.92; I^2^ = 93.53%, *p* < 0.001; Figure 9).

### Association of taking the combination of oral anti-diabetes and insulin with poor glycemic control

**Figure 10:**
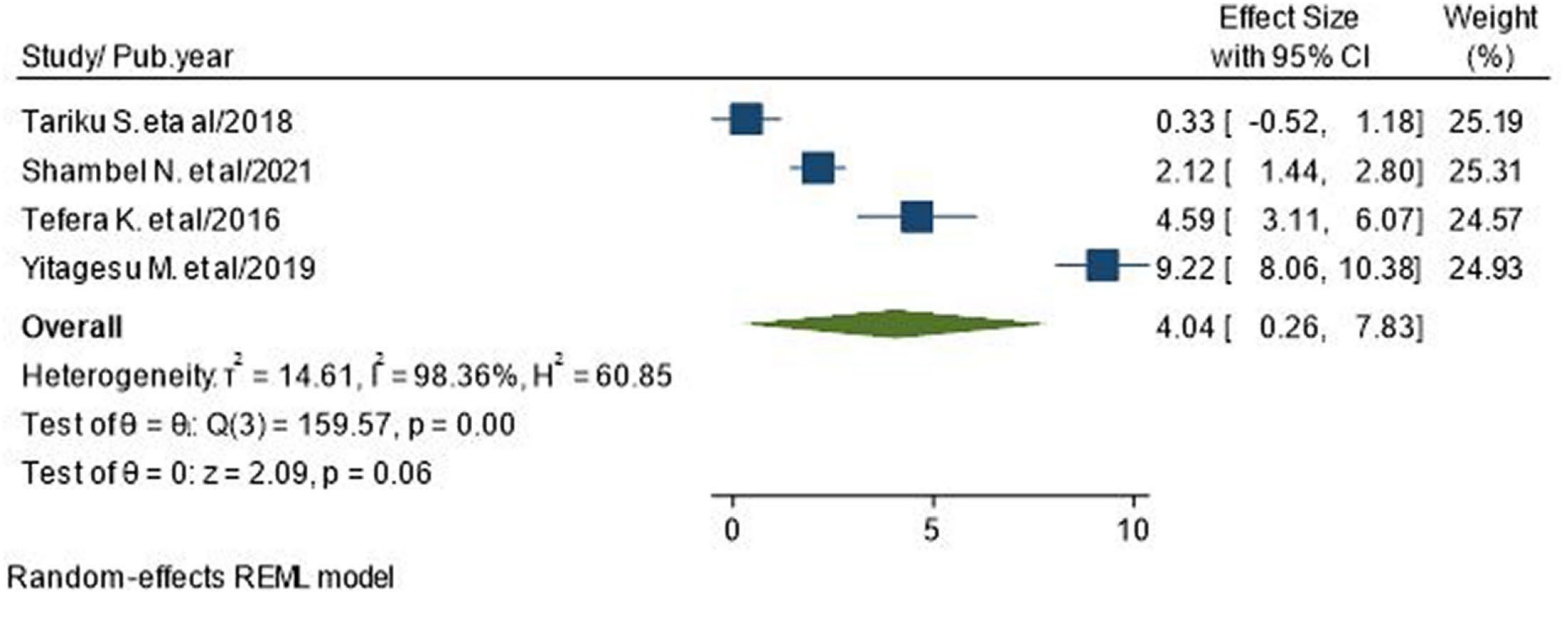
Association between low self-care adherence and poor glycemic control in type 2 diabetic patients.

Four studies with 1,474 participants reported the association between receiving a combination of oral anti-diabetic drugs and insulin and poor glycemic control in T2D patients (7, 19, 27, 36). The pooled odds ratio showed that there are no significant associations between receiving both oral anti-diabetics and insulin and poor glycemic control (AOR = 4.04; 95% CI: 0.26–7.83; I^2^ = 98.36%, *p* = 0.06; Figure 10).

## Discussion

In this study, we have conducted a comprehensive review to evaluate the pooled estimates and factors of poor glycemic control among T2D adults aged 18 years and older in Ethiopia. This systematic review and meta-analysis is, as far as we are aware the first meta-analysis on the prevalence and associated factors of poor glycemic control among people with T2D in Ethiopia. In this meta-analysis, 23 articles with a total of 6,643 study subjects were included. The overall prevalence of poor glycemic control among T2D in this study was 61.11% (95% CI: 57.14–65.19).

In accordance with our findings, a recent meta-analysis in the Middle East and North Africa reported 63% poor glycemic control in T2D (8). Similarly, a large cross-sectional study from Morocco revealed that 66.3% of T2D patients had poor glycemic control (37). A previous review on glycemic control for overall diabetes in Ethiopia also confirmed that 65.6% of patients had poor glycemic control based on fasting blood glucose measurement (9). On the contrary, our result is higher than a study reported in China (44%). The lower poor glycemic control prevalence observed in China might be due to higher literacy rates in industrialized nations, which likely leads to a better understanding of the disease (38). Having adequate knowledge about diabetes is essential for improving blood glucose and preventing acute and chronic complications.

The observed high heterogeneity of the studies included in this review could be largely attributed to a broad range of poor glycemic control prevalence across the studies. Thus, we further conducted subgroup analyses stratified by study region and publication year. In terms of region, Addis Ababa had the highest prevalence of poor glycemic control (68.57%). Addis Ababa is the capital city of the country, where economic growth and development are relatively higher compared to other areas of the country. Individuals residing in urban and industrialized areas often become physically inactive, consume highly processed and energy-rich foods, and experience stressful environments with inadequate sleep, which could all make controlling blood glucose difficult in T2D (39–41). Hence, healthcare professionals who manage diabetes should pay more attention to patients who live in towns to improve their blood glucose levels and overall health. With regard to subgroup analysis by publication year, we have observed a closer prevalence of poor glycemic control between studies published before 2019, which is 61.76, and 60.75% in 2019 and after.

Our findings show that older age, not attending formal education, longer duration with diabetes (>10 years), comorbidity, and low adherence to diabetes management were the factors contributing to poor glycemic control in T2D. Furthermore, this study demonstrated that older people >50 years were more likely to have poor glycemic control. This finding was supported by previous studies (15, 42, 43). In contrast, some studies have reported a contrary association between age and glycemic control. A previous review on glycemic control in diabetic patients reported that younger ages are more vulnerable to poor glycemic control (9). Therefore, future studies should consider focusing on age-specific factors for glycemic control.

Diabetes in people who had not attended formal education was also linked to poor glycemic control. This result is in line with previous meta-analyses in Ethiopia (9), a review in Gulf Cooperation Countries (44), and a study in Brazil (45). A low educational level leads to low health literacy, resulting in less knowledge about the disease and glycemic control (46). Hence, health professionals are advised to include a comprehensive health education program on glycemic control in their patient care routine for T2D.

The longer duration of diabetes, known to increase the risk of poor glycemic control, has been identified as a key factor contributing to poor glycemic control in diabetes (37). Our finding confirmed previous reports associating longer durations of diabetes with poor glycemic control. Odai Hamed et al. demonstrated that patients with longer durations of diabetes were associated with the occurrence of poor glycemic control (8). The unfavorable effect of a longer duration of diabetes on glycemic control could be possibly due to inadequate insulin secretion resulting from gradual dysfunction of the β-cell (47). Considering this, people with T2D should be classified as high risk due to the higher probability of losing control over their blood glucose levels. They require more specific and close attention to control blood glucose levels at the optimal level. In addition, our study revealed that T2D patients with comorbidities are more likely to poorly control their blood glucose, and this result is supported by a meta-analysis (48). Studies suggest that diabetes coupled with comorbidities has an adverse effect on the control of blood glucose in diabetes (49). This implies clinicians should design strategies emphasizing the management of diabetes with comorbidities. Low-level adherence is the other important factor leading to inadequate blood glucose control.

We found that T2D with a low level of adherence has 3.67 times higher odds of poor glycemic control compared to diabetes with good glycemic control. Low-level adherence has become a popular challenge over the past few years, which could have a paramount contribution to poor glycemic control in diabetes. Previous studies also mentioned low adherence as a factor in poor glycemic control (9, 50). It is highly recommended that greater effort be focused on counseling and improving adherence rather than changing medications and adjusting the dose (51). Another study showed that improving adherence results in better glycemic control, and better glycemic control was observed in highly adherent patients than in non-adherent patients (52). This indicates that activities such as health education and continuous glycemic control that improve adherence levels are priorities. Overall, the result implies that policymakers, health professionals, and concerned bodies should encourage better control of blood glucose to reduce complications related to poor glycemic control and improve the overall quality of life.

## Conclusion

To conclude, our study indicated a high prevalence of poor glycemic control. Factors such as older age, not attending formal education, longer duration of diabetes, comorbidity, and low adherence were associated with poor glycemic control. As diabetes is a progressive chronic disease, complications arise from inadequate diabetes management. This is an issue of concern, as these patients face more challenges in controlling the disease process, which might lead to the worst health outcome. To that end, health professionals should be aware of the need for special emphasis on specific groups such as older diabetics, individuals with low educational status, those with a longer duration of diabetes, diabetes with comorbidities, and those who are not adherent to treatments. One way is to evaluate all diabetes patients with respect to the individualized needs of glycemic targets, lifestyles, and the medications needed to achieve those targets. Efforts to lower A1c should be carried out considering patient characteristics such as age, educational status, duration of diabetes mellitus, comorbidity, and adherence to ensure long-term glycemic control. Further longitudinal cohort studies evaluating the prevalence and factors of poor glycemic control among T2D are required.

### Strength and limitations

This study has some limitations. First, articles were restricted to only the English language, which could potentially exclude other relevant articles. Second, the meta-analyses showed high heterogeneity in the pooled prevalence. Sample size variations across studies, different geographical areas, and other unknown factors in the studies might explain the high heterogeneity of estimates. Third, compared to other articles, two articles have used FPG > 154 mg/dL as screening criteria for poor glycemic control; this may have an influence on the overall estimates. Despite all these limitations, this study also has strengths. Several factors associated with poor glycemic control were identified. In addition, the authors followed systematic approaches to include more studies and maintain the quality of the study.

## Data availability statement

The original contributions presented in the study are included in the article/Supplementary material, further inquiries can be directed to the corresponding author/s.

## Author contributions

KT: Conceptualization, Formal analysis, Investigation, Methodology, Software, Writing – original draft, Writing – review & editing. NG: Conceptualization, Supervision, Investigation, Validation, Writing – review & editing. LY: Methodology, Validation, Writing – review & editing. YY: Project administration, Validation, Writing – review & editing. MK: Software, Supervision, Validation, Writing – review & editing.
