# Supplementary material for: Determinants of poor glycemic control among type 2 diabetes in Ethiopia: a systematic review and meta-analysis
Source: Front Public Health. 2024 Feb 5;12:1256024. doi: 10.3389/fpubh.2024.1256024 (PMC10876054; doi:10.3389/fpubh.2024.1256024)
Supplement: Supplementary file 1 [file Table_1.docx]

# S 1 Table

#
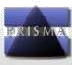
PRISMA 2020 Checklist

|  |  |  |  |  |  |
| --- | --- | --- | --- | --- | --- |
|  |  | **Item** | **Checklist item** |  | **Location** |
| Section and |  |  |  |  | **where item** |
|  |  | **#** |  |  |  |
|  |  |  |  |  | **is reported** |
| Topic |  |  |  |  |  |
| **TITLE** | |  |  |  |  |
| Title |  | 1 | Identify the report as a systematic review. |  | 1 |
| **ABSTRACT** | |  |  |  |  |
| Abstract |  | 2 | See the PRISMA 2020 for Abstracts checklist. |  | 1 |
| **INTRODUCTION** | |  |  |  |  |
| Rationale | 3 | | Describe the rationale for the review in the context of existing knowledge. | 2 | |
| Objectives | 4 | | Provide an explicit statement of the objective(s) or question(s) the review addresses. | 2 | |
| **METHODS** | |  |  |  |  |
| Eligibility criteria | 5 | | Specify the inclusion and exclusion criteria for the review and how studies were grouped for the syntheses. | 2 | |
| Information | 6 | | Specify all databases, registers, websites, organizations, reference lists and other sources searched or consulted to identify studies. Specify the | 2 | |
| sources |  |  | date when each source was last searched or consulted. |  |  |
| Search strategy | 7 | | Present the full search strategies for all databases, registers and websites, including any filters and limits used. | 2 | |
| Selection process | 8 | | Specify the methods used to decide whether a study met the inclusion criteria of the review, including how many reviewers screened each record | 3 | |
|  |  |  | and each report retrieved, whether they worked independently, and if applicable, details of automation tools used in the process. |  |  |
| Data collection | 9 | | Specify the methods used to collect data from reports, including how many reviewers collected data from each report, whether they worked | 3 | |
| process |  |  | independently, any processes for obtaining or confirming data from study investigators, and if applicable, details of automation tools used in the |  |  |
|  |  |  | process. |  |  |
| Data items | 10a | | List and define all outcomes for which data were sought. Specify whether all results that were compatible with each outcome domain in each | 2 | |
|  |  |  | study were sought (e.g. for all measures, time points, analyses), and if not, the methods used to decide which results to collect. |  |  |
|  | 10b | | List and define all other variables for which data were sought (e.g. participant and intervention characteristics, funding sources). Describe any |  | 2 |
|  |  |  | assumptions made about any missing or unclear information. |  |  |
| Study risk of bias | 11 | | Specify the methods used to assess risk of bias in the included studies, including details of the tool(s) used, how many reviewers assessed each | 2 | |
| assessment |  |  | study and whether they worked independently, and if applicable, details of automation tools used in the process. |  |  |
| Effect measures | 12 | | Specify for each outcome the effect measure(s) (e.g. risk ratio, mean difference) used in the synthesis or presentation of results. |  |  |
|  |  |  |  |  | 3 |
| Synthesis | 13a | | Describe the processes used to decide which studies were eligible for each synthesis (e.g. tabulating the study intervention characteristics and |  | 3 |
| methods |  |  | Comparing against the planned groups for each synthesis (item #5)). |  |  |
|  | 13b | | Describe any methods required to prepare the data for presentation or synthesis, such as handling of missing summary statistics, or data |  | 3 |
|  |  |  | Conversions. |  |  |
|  | 13c | | Describe any methods used to tabulate or visually display results of individual studies and syntheses. | 3 | |
|  |  |  |  |  | |
|  | 13d | | Describe any methods used to synthesize results and provide a rationale for the choice(s). If meta-analysis was performed, describe the | 3 | |
|  |  |  | model(s), method(s) to identify the presence and extent of statistical heterogeneity, and software package(s) used. |  |  |
|  | 13e | | Describe any methods used to explore possible causes of heterogeneity among study results (e.g. subgroup analysis, meta-regression). | 3 | |

|  | 13f | Describe any sensitivity analyses conducted to assess robustness of the synthesized results. | 3 |
| --- | --- | --- | --- |


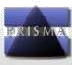
**PRISMA 2020 Checklist**

|  |  |  |  |  |  |
| --- | --- | --- | --- | --- | --- |
|  |  | **Item** | **Checklist item** |  | **Location** |
| Section and |  |  |  |  | **where item** |
|  |  | **#** |  |  |  |
|  |  |  |  |  | **is reported** |
| Topic |  |  |  |  |  |
| Reporting bias | 14 | | Describe any methods used to assess risk of bias due to missing results in a synthesis (arising from reporting biases). |  | 2 |
| assessment |  |  |  |  |  |
| Certainty | 15 | | Describe any methods used to assess certainty (or confidence) in the body of evidence for an outcome. |  | Not- |
| assessment |  |  |  |  | applicable |
| **RESULTS** | |  |  |  |  |
| Study selection | 16a | | Describe the results of the search and selection process, from the number of records identified in the search to the number of studies included in | 3 | |
|  |  |  | the review, ideally using a flow diagram. |  |  |
|  | 16b | | Cite studies that might appear to meet the inclusion criteria, but which were excluded, and explain why they were excluded. | 4 | |
| Study | 17 | | Cite each included study and present its characteristics. | 3 | |
| characteristics |  |  |  |  |  |
| Risk of bias in | 18 | | Present assessments of risk of bias for each included study. | 4 | |
| studies |  |  |  |  |  |
| Results of | 19 | | For all outcomes, present, for each study: (a) summary statistics for each group (where appropriate) and (b) an effect estimate and its precision |  | 5 |
| individual studies |  |  | (e.g. confidence/credible interval), ideally using structured tables or plots. |  |  |
| Results of | 20a | | For each synthesis, briefly summarize the characteristics and risk of bias among contributing studies. | 5 | |
| syntheses |  |  |  |  |  |
|  | 20b | | Present results of all statistical syntheses conducted. If meta-analysis was done, present for each the summary estimate and its precision (e.g. |  | 3 |
|  |  |  | confidence/credible interval) and measures of statistical heterogeneity. If comparing groups, describe the direction of the effect. |  |  |
|  | 20c | | Present results of all investigations of possible causes of heterogeneity among study results. | 3 | |
|  | 20d | | Present results of all sensitivity analyses conducted to assess the robustness of the synthesized results. | 14 | |
| Reporting biases | 21 | | Present assessments of risk of bias due to missing results (arising from reporting biases) for each synthesis assessed. |  | 4 |
|  |  |  |  |  |  |
| Certainty of | 22 | | Present assessments of certainty (or confidence) in the body of evidence for each outcome assessed. |  | Not- |
| evidence |  |  |  |  | applicable |
| **DISCUSSION** | |  |  |  |  |
| Discussion | 23a | | Provide a general interpretation of the results in the context of other evidence. | 7 | |
|  | 23b | | Discuss any limitations of the evidence included in the review. | 9 | |
|  | 23c | | Discuss any limitations of the review processes used. | 9 | |
|  | 23d | | Discuss implications of the results for practice, policy, and future research. | 9 | |
| **OTHER INFORMATION** | | |  |  |  |
| Registration and | 24a | | Provide registration information for the review, including register name and registration number, or state that the review was not registered. |  | 2 |
| protocol |  |  |  |  |  |
|  | 24b | | Indicate where the review protocol can be accessed, or state that a protocol was not prepared. |  | 2 |
|  |  |  |  |  |  |
|  | 24c | | Describe and explain any amendments to information provided at registration or in the protocol. |  | Not- |
|  |  |  |  |  | applicable |


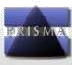
**PRISMA 2020 Checklist**

|  |  |  |  |  |  |  |
| --- | --- | --- | --- | --- | --- | --- |
|  |  | **Item** |  | **Checklist item** |  | **Location** |
| Section and |  |  |  |  |  | **where item** |
|  |  | **#** |  |  |  |  |
|  |  |  |  |  |  | **is reported** |
| Topic |  |  |  |  |  |  |
| Support | 25 | | Describe sources of financial or non-financial support for the review, and the role of the funders or sponsors in the review. | |  | 10 |
|  |  |  |  |  |  |  |
| Competing | 26 | | Declare any competing interests of review authors. | | 10 | |
| interests |  |  |  |  |  |  |
| Availability of | 27 | |  | Report which of the following are publicly available and where they can be found: template data collection forms; data extracted from included | Not applicable | |
| data, code and |  |  |  | studies; data used for all analyses; analytic code; any other materials used in the review. |  |  |
| other materials |  |  |  |  |  |  |

*From:* Page MJ, McKenzie JE, Bossuyt PM, Boutron I, Hoffmann TC, Mulrow CD, et al. The PRISMA 2020 statement: an updated guideline for reporting systematic reviews. BMJ 2021;372:n71. doi: 10.1136/bmj.n71

For more information, visit: <http://www.prisma-statement.org/>
