# Supplementary material for: Determinants of poor glycemic control among type 2 diabetes in Ethiopia: a systematic review and meta-analysis
Source: Front Public Health. 2024 Feb 5;12:1256024. doi: 10.3389/fpubh.2024.1256024 (PMC10876054; doi:10.3389/fpubh.2024.1256024)
Supplement: Supplementary file 2 [file Table_2.DOCX]

S 2 Table. Quality assessment for the included Studies

| Item | The criteria for inclusion in the sample clearly defined | Describe study setting and participant | Valid and reliable exposure measurement | Objective and standard criteria for measurement | Identified confounder | Strategies to deal with confounders | Valid and reliable outcome measurement | Appropriate statically analysis | No of ‘yes’ ‘ |
| --- | --- | --- | --- | --- | --- | --- | --- | --- | --- |
| Alebachew F. et al | Yes | Yes | No | Yes | Yes | No | Yes | Yes | 6/8=75 |
| Alemayehu D. et al | Yes | Yes | Yes | Yes | No | No | Yes | Yes | 6/8=75 |
| Amass S. et al | Yes | Yes | No | Yes | Yes | No | Yes | Yes | 6/8=75 |
| Asnakew A. et al | Yes | Yes | No | Yes | Yes | Yes | Yes | Yes | 7/8=87.5 |
| Bayisa B. et al | Yes | Yes | No | Yes | Yes | Yes | Yes | Yes | 7/8=87.5 |
| Berhane F. et al | Yes | Yes | Yes | Yes | Yes | No | Yes | Yes | 7/8=87.5 |
| Daba A. et al | Yes | Yes | Yes | Yes | No | No | Yes | Yes | 6/8=75 |
| Daniel M. et al | Yes | Yes | No | Yes | Yes | Yes | Yes | No | 6/8=75 |

| Gebre T. et al | Yes | Yes | Yes | Yes | No | Yes | Yes | No | 6/8=75 |
| --- | --- | --- | --- | --- | --- | --- | --- | --- | --- |
| Ginenus F. et al | Yes | yes | No | Yes | Yes | Yes | Yes | Yes | 7/8=87.5 |
| Gudisa B. et al | Yes | Yes | Yes | No | Yes | No | Yes | Yes | 6/8=75 |
| Minyahil A. et al | Yes | Yes | Yes | Yes | Yes | Yes | Yes | No | 5/8=62.5 |
| Nasir T. et al | Yes | Yes | Yes | Yes | Yes | Yes | No | Yes | 7/8=87.5 |
| Nigussie G. et al | Yes | Yes | No | Yes | No | Yes | Yes | Yes | 6/8=75 |
| Rodas G. et al | Yes | Yes | Yes | Yes | Yes | Yes | Yes | No | 7 /8=87.5 |
| Shambel N. et al | Yes | Yes | No | Yes | Yes | No | Yes | Yes | 6/8=75 |
| Tadele E. et al | Yes | Yes | Yes | Yes | Yes | Yes | Yes | No | 7 /8=87.5 |
| Tadesse A. et al | Yes | Yes | Yes | Yes | Yes | Yes | Yes | No | 7 /8=87.5 |
| Tariku S. et al | Yes | Yes | No | Yes | Yes | No | Yes | Yes | 6/8=75 |
| Tefera K. et al | Yes | Yes | Yes | Yes | Yes | Yes | Yes | No | 7 /8=87.5 |
| Tewodros Y. et al | Yes | Yes | Yes | Yes | No | Yes | Yes | Yes | 7/8=87.5 |
| Yohannes T. et al | Yes | Yes | No | Yes | Yes | Yes | Yes | Yes | 7/8=87.5 |
| Yitagesu M. et al | Yes | Yes | Yes | Yes | Yes | No | Yes | Yes | 7/8=87.5 |
