# Supplementary material for: Determinants of poor glycemic control among type 2 diabetes in Ethiopia: a systematic review and meta-analysis
Source: Front Public Health. 2024 Feb 5;12:1256024. doi: 10.3389/fpubh.2024.1256024 (PMC10876054; doi:10.3389/fpubh.2024.1256024)
Supplement: Supplementary file 3 [file Table_3.DOCX]

S 3 Table. Risk of bias of assessment for the cross-sectional studies

| Item | External validity | | | | Internal validity | | | | | |  | |
| --- | --- | --- | --- | --- | --- | --- | --- | --- | --- | --- | --- | --- |
|  | Representativeness s of the target population | Representativeness s of the sampling frame | Radom samplin g or census | Minimal responses e bias | Data were collect d directly | Acceptable e case definition used in the study | Valid and reliable measurement t | The same mode of data collection n for all study subject | Appropriate e length of prevalence period for parameter of interest | Appropriate numerators and denominator s of interest | No of yes | **Summ ary of risk of bias** |
| Alebachew F. et al | Yes | Yes | No | Yes | Yes | No | Yes | Yes | Yes | Yes | 8 | Low-  risk |
| Alemayehu D. et al | Yes | Yes | Yes | Yes | No | No | Yes | Yes | Yes | Yes | 8 | Loiw risk |
| Amass S. et al | Yes | Yes | No | Yes | Yes | No | Yes | Yes | Yes | Yes | 8 | Low- risk |
| Asnakew A. et al | Yes | Yes | No | Yes | Yes | Yes | Yes | Yes | Yes | Yes | 9 | Low – risk |
| Bayise B. et al | Yes | Yes | No | Yes | Yes | Yes | Yes | Yes | Yes | Yes | 9 | Low- risk |
| Berhane F. et al | Yes | Yes | Yes | Yes | Yes | No | Yes | Yes | Yes | Yes | 9 | Low- risk |
| Daba A. et al | Yes | Yes | No | Yes | Yes | Yes | Yes | Yes | Yes | Yes | 9 | Low- risk |
| Daniel M. et al | Yes | Yes | Yes | Yes | No | No | Yes | Yes | Yes | Yes | 8 | Low- risk |
| Gebre T. et al | Yes | Yes | No | Yes | Yes | Yes | Yes | No | Yes | Yes | 8 | Low- risk |

| Ginenus F. et al | Yes | Yes | Yes | Yes | No | Yes | Yes | No | Yes | Yes | 8 | Low- risk |
| --- | --- | --- | --- | --- | --- | --- | --- | --- | --- | --- | --- | --- |
| Gudisa B. et al | Yes | Yes | Yes | Yes | No | No | Yes | Yes | Yes | Yes | 8 | Low-  risk |
| Minyahil A. et al | Yes | yes | No | Yes | Yes | No | Yes | No | Yes | Yes | 7 | Moderate- risk |
| Nasir T. et al | Yes | Yes | Yes | No | Yes | No | Yes | Yes | Yes | Yes | 8 | Low-  risk |
| Nigussie G. et al | Yes | Yes | Yes | Yes | Yes | Yes | Yes | No | Yes | Yes | 9 | Low-  risk |
| Rodas G. et al | Yes | Yes | Yes | Yes | Yes | Yes | No | Yes | Yes | Yes | 8 | Low- risk |
| Shambel N. et al | Yes | Yes | No | Yes | Yes | Yes | Yes | Yes | Yes | Yes | 9 | Low risk |
| Tadele E. et al | Yes | Yes | Yes | Yes | Yes | Yes | Yes | Yes | No | No | 8 | Low- risk |
| Tadesse A. et al | Yes | Yes | Yes | Yes | Yes | Yes | Yes | No | No | Yes | 8 | Low- risk |
| Tariku S. et al | Yes | Yes | No | Yes | Yes | Yes | Yes | Yes | Yes | Yes | 9 | Low risk |
| Tefera K. et al | Yes | Yes | Yes | No | Yes | No | Yes | Yes | Yes | Yes | 8 | Low-  risk |
| Tewodros Y. et al | Yes | Yes | Yes | Yes | No | Yes | Yes | Yes | No | Yes | 8 | Low-  risk |
| Yohannes T. et al | Yes | Yes | Yes | Yes | Yes | Yes | Yes | No | Yes | Yes | 9 | Low-  risk |
| Yitagesu M. et al | Yes | Yes | No | Yes | Yes | Yes | No | Yes | Yes | Yes | 8 | Low-risk |
